# Neurophysiological measurements of planarian brain activity: a unique model for neuroscience research

**DOI:** 10.1242/bio.060480

**Published:** 2024-08-08

**Authors:** Orel Benita, Nir Nesher, Tal Shomrat

**Affiliations:** ^1^ Department of Neurobiology, Hebrew University, Jerusalem 9190401, Israel; ^2^ Faculty of Marine Sciences, Ruppin Academic Center, Michmoret 4029700, Israel

**Keywords:** Planaria, Brain, Electrophysiology, Multi-unit recording, Two-headed worm, Model organism

## Abstract

Planarians are well-known model organisms for regeneration and developmental biology research due to their remarkable regenerative capacity. Here, we aim to advocate for the use of planaria as a valuable model for neurobiology, as well. Planarians have most of the major qualities of more developed organisms, including a primal brain. These traits combined with their exceptional regeneration capabilities, allow neurobiological experiments not possible in any other model organism, as we demonstrate by electrophysiological recording from planaria with two heads that controlling a shared body. To facilitate planarian neuroscience research, we developed an extracellular multi-unit recording procedure for the planarians fragile brain (*Dugesia japonica*). We created a semi-intact preparation restrained with fine dissection pins, enabling hours of reliable recording, via a suction electrode. Here, we demonstrate the feasibility and potential of planarian neurophysiological research by characterizing the neuronal activity during simple learning processes and responses to various stimuli. In addition, we examined the use of linalool as anesthetic agent to allows recordings from an intact, large worm and for fine electrophysiological approaches such as intracellular recording. The demonstrated ability for neurophysiological measurements, along with the inherent advantages of planarians, promotes this exceptional model organism for neuroscience research.

## Teaser

**Summary:** The first recording of well-defined extracellular multiunit activity from the freshwater planarian brain, obtained by a straight-forward technique which promoting the planarian as a unique model organism for neuroscience research.

## INTRODUCTION

The fresh water planarians share many characteristics with more advanced organisms (Sánchez Alvarado, 2004). Despite their body complexity, planaria possess one of the most extraordinary regeneration capabilities in nature, whereby a completely new worm can regenerate from almost any fragment of the original worm, in less than 2 weeks (although, not all planarian species display the same regenerative capabilities; Vila-Farré et al., 2023). These features have placed the planarian as a leading model organism for regeneration and developmental biology research (Ivankovic et al., 2019; Newmark and Sánchez Alvarado, 2022). Moreover, it is emerging as an important model for environmental toxicology and pharmacology (Ireland and Collins, 2022; Pagán, 2017; Wu and Li, 2018).

The purpose of this work is to establish planaria as an exceptional model for neuroscience research as well. Planaria combine a primal brain (Pagán, 2019) composed of bilateral cephalic ganglions that contain several thousand neurons, with vertebrate features such as multipolar neurons, dendritic spines, and containing most of the neurotransmitters and neuromodulators found in vertebrates’ nervous systems (Buttarelli et al., 2008; Sarnat and Netsky, 1985). They also possess relatively sophisticated sensory capabilities, such as detecting intensity and direction of light through a pair of primitive eyes (Paskin et al., 2014), chemical gradients (Mason, 1975; Miyamoto and Shimozawa, 1985) and vibration (Fulgheri and Messeri, 1973). These perception mechanisms are integrated via the worm's nervous system into a rich and complex set of behaviors such as environmental familiarization (Shomrat and Levin, 2013), drug withdrawal (Pagán, 2017), and decision-making (Inoue et al., 2015). The exceptional regeneration capabilities allow neurobiological experiments not possible in any other animal model. For example, it is the only organism that can easily be manipulated to form a functional animal with double heads, allowing the investigation of how these two separated brains are integrating and control a shared body (Hull, 1940). Another example is brain transplantation (Davies et al., 1985; Reddien and Sánchez Alvarado, 2004; Rojo-Laguna et al., 2019). Lastly, because planaria can easily regrow an entire brain, it was first model used in the 1960s for investigating the controversial theory about molecular encoding of memory. Nowadays, this revolutionary hypothesis is gaining renewed interest with the current knowledge about the potential role of epigenetic elements in memory storage. (Blackiston et al., 2015; Bédécarrats et al., 2018; Dias and Ressler, 2014; Neuhof et al., 2016; Smalheiser et al., 2001).

Electrophysiology is a fundamental approach for any neurobiological research. The gross structure of the planaria brain and peripheral nervous system is relatively well-documented (Agata and Umesono, 2008; Okamoto et al., 2005) however, only few neurophysiological studies have been conducted to uncover the mechanisms behind the worms’ behavior (Aoki et al., 2009; Azuma et al., 1994; Freiberg et al., 2023). During the 1970s and early 80s there was some electrophysiological research on marine flatworms (Koopowitz, 1975; Solon and Koopowitz, 1982). However, compared to freshwater planarians the maintenance of marine flatworms in the lab is much harder, their regenerative abilities are significantly inferior, and the extent of scientific knowledge on them is limited. There are two main factors that make electrophysiological measurements challenging. First, the loosely formed, very fragile and tiny planarian brain with no surrounding hard tissue, makes it difficult to achieve durable and precise fixation of the recording micropipette or wire electrode (Okamoto et al., 2005). The second obstacle is the lack of an efficient anesthetic agent to allow fine *in-vivo* electrophysiological recordings (Stevenson and Beane, 2010). Many protocols have been attempted to date with the goal of immobilizing the planarian (Gumbrys, 2017). Some achieved partial immobility that allowed fine microscopy approaches. In other protocols, immobilization has been achieved with the use of mechanical restraint but that accomplished with a physical barrier that makes it difficult for electrophysiology (Dexter et al., 2014; Guedelhoefer and Sánchez Alvarado, 2012; Jaenen et al., 2023). Efficient anesthetic procedure for electrophysiology should completely immobilize the worm without abolishing its brain activity.

The planarian unlimited regeneration capability with the feasibility of fine electrophysiological experiments, has the potential to make an exceptional contribution to the field of neuroscience (Omond and Lesku, 2023). Here, we made an attempt to address both obstacles described above. We gained high-quality extracellular multi-unit recording with suction electrodes and examined procedures for restraining worms with fine dissection pins and linalool as an anesthetic.

## RESULTS

### The neuronal response profiles to electric shock, ultraviolet light, and vibration

Two aversive stimuli, ultraviolet (UV) light and electric shock, in addition to non-aversive, mild vibration stimulation were examined in a half-head preparation. Each stimulus induces a different activity profile.

The evoked activity from the UV light occurs with a latency of ∼200 ms (Figs 3E,G and 4B,D). The ∼200 ms latency appeared for any UV-light stimulus duration (even less than 50 ms, data not shown) as long as it induced clear and robust evoked activity.

**Fig. 1.**
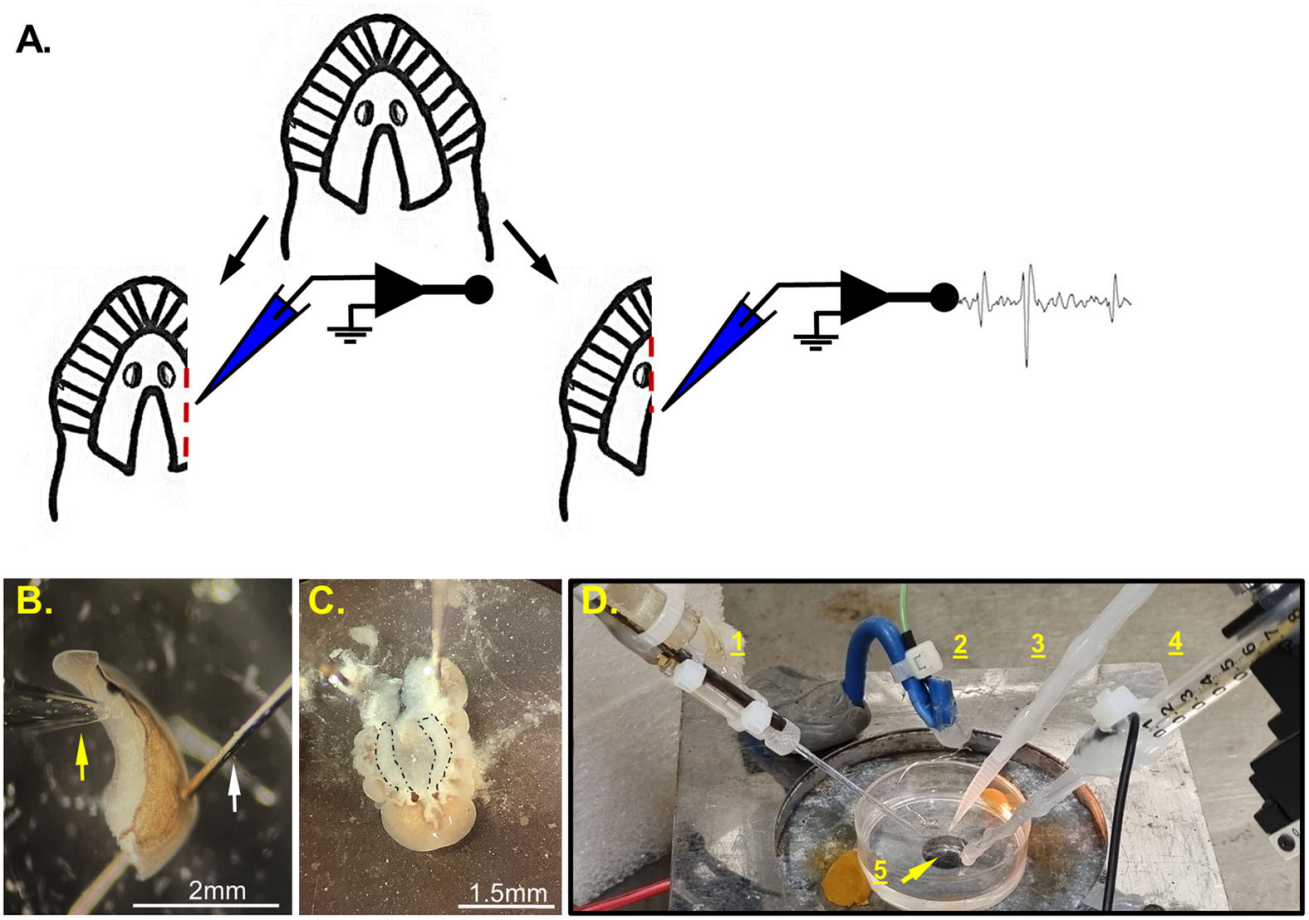
**The semi-intact preparation and the experimental setup.** (A) Diagram illustrates the structure of a planaria head, which includes the two ‘hemispheres’ of the brain with the lateral branches, and the location of the eye spots (ocelli). Below, are the two cutting planes used in this work (according to the region of interest), left: ¾ head preparation; right: a half head preparation. (B) Half head preparation held by a pin (white arrow) with the suction electrode (yellow arrow) connected at the brain region located just posterior to the eye. (C) A different possible approach to expose the brain for electrophysiological recording (not used for the experiments presented in this work). The brain is uncovered by dorsally peeling the skin, muscles and gastrovascular layers. The two brains ‘hemispheres’ emphasized by dashed black line. (D) Photograph of the recording chamber setup showing the petri dish with transparent SYLGARD 184 silicone bottom. (1) the suction electrode, (2) the reference electrode made of platinum-iridium coil, (3) the plastic rod that delivers the vibration stimulus, (4) the bipolar platinum-iridium stimulation electrode, (5) the hole in the recording stage through which the ultraviolet light is projected toward the worm that is pinned above (the worm is not shown).

**Fig. 2.**
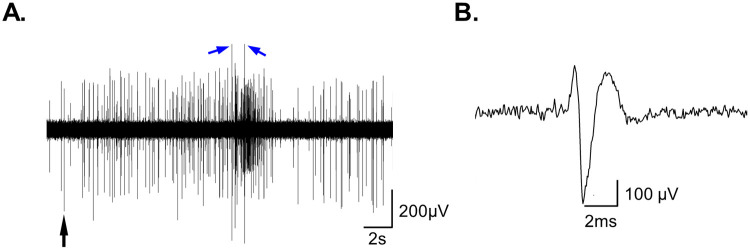
**A representative example of the extracellular multiunit recording.** The extracellular multi-unit recording differentially amplified (×10k) and bandpass-filtered (0.3–10 kHz). (A) Spontaneous neuronal activity followed by an evoked response to 1 s projected ultraviolet light (∼390 nm). The blue arrows indicate the electric artifacts of the 1 s light ON/OFF. (B) Enlargement of the spike labeled by black arrow in ‘A’.

**Fig. 3.**
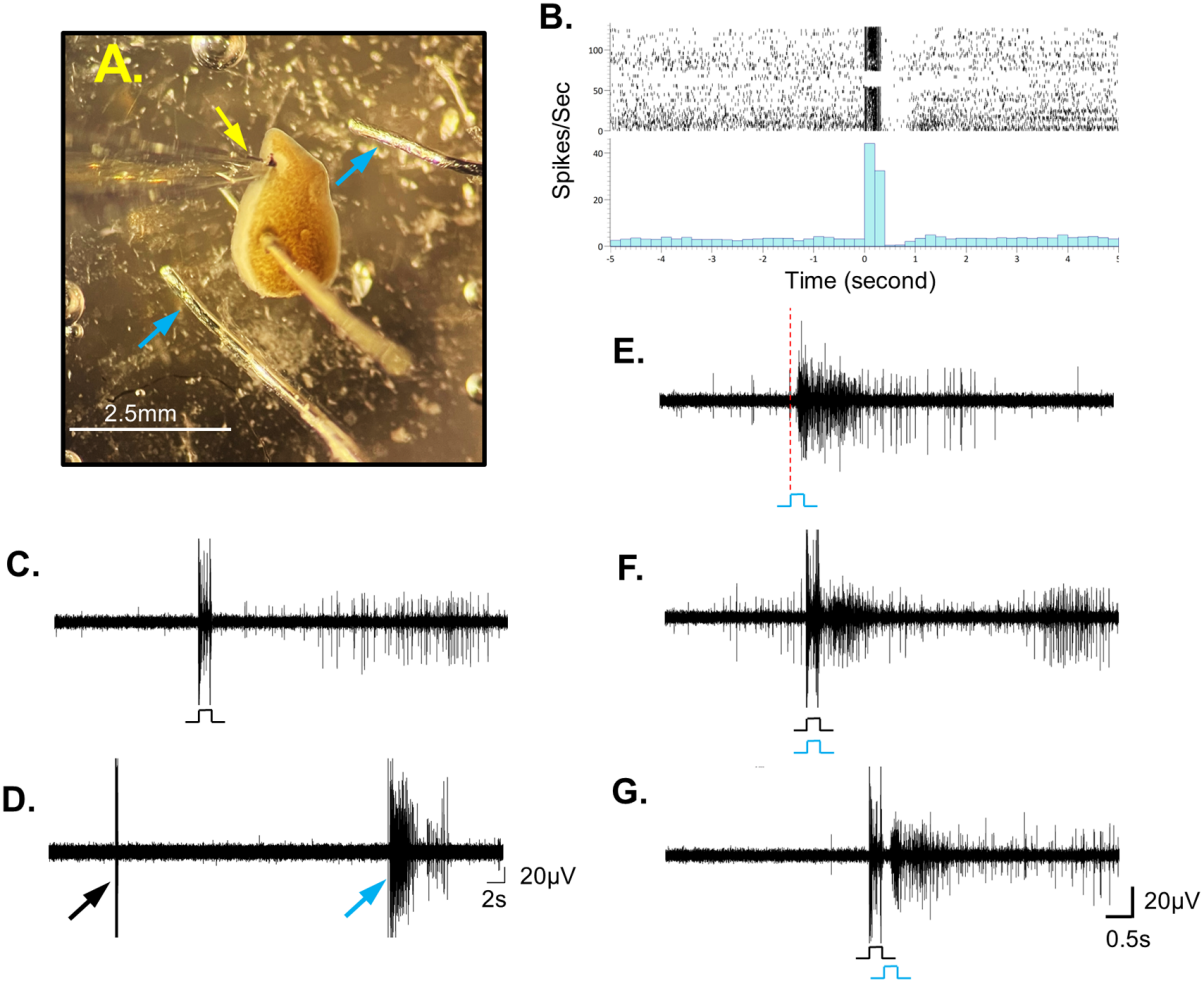
**The neuronal activity response profile to electric shock stimulus.** (A) The half-head preparation, from which the shown responses to the electric shock stimulus were recorded. The blue arrows indicate the platinum-iridium wire electrodes used to generate the electric shock stimulation. The yellow arrow highlights the suction electrode. (B) Raster plot and its peristimulus time histogram (spike rate, averaged in 200 ms bins) from recording session containing two blocks (∼25 min) of electric shock stimulation of 1 mA/250 ms with an inter trial interval of 30 s and a 10 min break between the blocks. Note that during the 250 ms stimulation there is a large artifact that sums up with the evoked activity to two large bins. Next, there is almost 1 s pause in activity followed by clear evoked activity that diminishes after few trials, coinciding with an elongation of the pause in activity. (C) The activity evoked by an electric shock, characterized by approximately 1 s pause and a delayed response occurring a few seconds after the stimuli. (D) Electric shock stimulation, where the evoked response has been abolished and only the stimulus artefact remained (black arrow), while there is still clear evoked response to aversive ultraviolet light (light blue arrow, 250 ms). (E) The activity evoked by aversive ultraviolet light with a latency of approximately 200 ms from the onset of the stimulus (indicated by the dashed red line). (F) Combination of simultaneous electric shock and ultraviolet light stimulations (both for 250 ms). The evoked activity of the ultraviolet light overcomes the pause typically induced by the electric shock. (G) When the ultraviolet light stimulation is applied immediately after the electric shock stimulation, the evoked activity overcomes the pause induced by the electric shock, but now with the 200 ms response latency, characterizing the light stimulus.

**Fig. 4.**
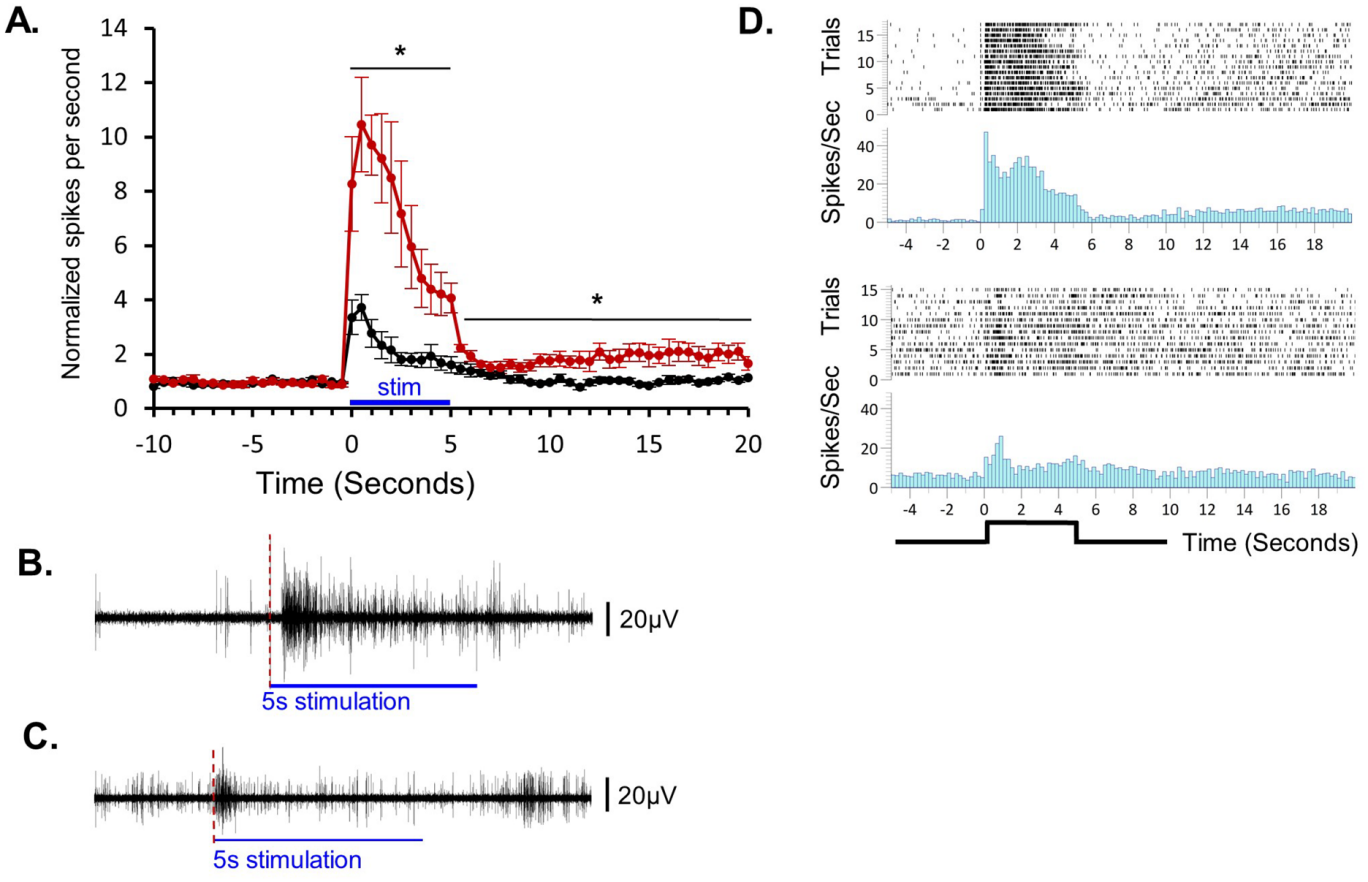
**Characterization of the sensory adaptation profile from repetitive stimulations of aversive ultraviolet light or mild vibration.** (A) Summary of experiments of brain activity recorded from half head preparations during vibration stimulus (black line, *N*=10) in comparison to the average response from experiments of brain activity during strong ultraviolet light stimulation (red line, *N*=8). Both stimuli were given for 5 s (stim) with 90 s inter trial interval. The Y axis: before averaging all the experiments together, each of the experiments data points (spike per second for 0.5 s bin) were normalized to the average spontaneous activity during the 30 s before the onset of the stimulus. The error bars represent the ±s.e.m; * repeated measure ANOVA, *P*<0.01. (B) an example of one trace with ultraviolet light stimulation. Note the ∼200 ms latency from the start of the light (red dashed line) and the evoked activity. (C) Similar to B, an example of a single trace with vibration stimulation. (D) Raster plots and their peristimulus time histogram (spike rate, averaged in 200 ms bins). The upper panel presenting the recorded responses for ultraviolet light stimulation. The lower panel presents the recording response for vibration stimulus. Note that in the upper panel histogram for the ultraviolet light stimulation, the first bin (200 ms) after the onset of the stimulus is a sum up of the artifact signals while the neuronal response occurs after ∼200 ms summed up at the next bin.

The electric shock (1 mA/250 ms), first causes a large artifact, which makes it impossible to trace the neuronal activity during the stimulus period. In most of the experiments, at the end of the stimulus, not only was there no evoked activity - but there was a long pause in spontaneous activity for several hundred milliseconds (Fig. 3B,C). In some of the experiments the pause ended with an elevated activity, compared to the activity before the stimulus (Fig. 3C). Moreover, that evoked activity abolishes after several trials (Fig. 3B,D) while behaviorally, the worms continued to react to the electric stimulation with contractions, even when there was no evoked brain activity (Fig. 3D). Interestingly, while the pause period suppressed the electric-shock-evoked activity as well as the spontaneous activity, it did not prevent the evoked activity following the UV-light stimulus, when both stimuli were applied simultaneously (Fig. 3F). When the UV light was applied immediately after the electrical stimulation, the robust evoked activity appeared with the typical ∼200 ms latency (Fig. 3G).

The vibration-stimuli-triggered activity is significantly less intense than that induced by the aversive UV light (Fig. 4, repeated measure ANOVA, *P*<0.01). In contrast to the UV-light stimulus, the vibration induces an inconsistent response latency from the initiation of the stimulus. In most of the recording sessions there was no latency at all (Fig. 4C,D), and in some sessions there was latency of tens to few hundred milliseconds. In a number of the experiments the latency periods increased slowly during the stimulation session, probably, as part of habituation process as described below.

### Characterization of sensory adaptation

Fig. 4 presents a comparative summary of the sensory adaptation to 5 s (90 s inter trial interval) of mild vibration stimulus (*n*=10) versus an aversive UV-light stimulation (*n*=8). The measurements of evoked activity were conducted on a half-head preparation. Both stimuli evoked responses exhibited a strong decrease that started after the first second. However, at the end of the stimulation, the response to the vibration ceased and returned to the baseline level prior to stimulation, while the activity following the UV-light maintained for tens of seconds in a slightly higher sensitization level of 1.5 to 2-fold above the baseline level before stimulation.

### The learning processes habituation and dishabituation

**Fig. 5.**
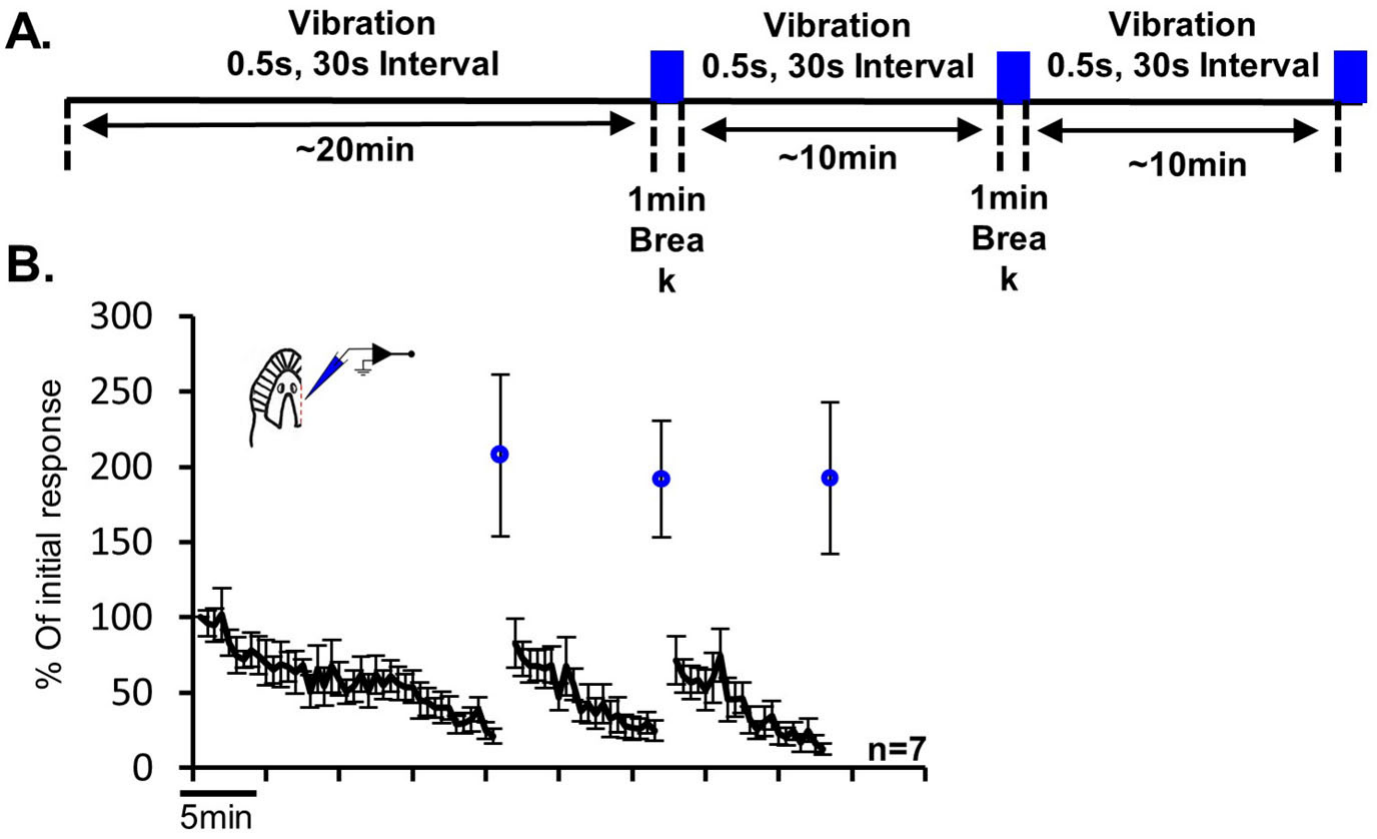
**Brain activity recorded from ¾ head preparation during habituation and dishabituation.** (A) The behavioral protocol: three blocks of ∼20, ∼10 and ∼10 min of habituation to 0.5 s vibration stimulus 30 s inter trial interval. Each block ends with 1 s ultraviolet light that serves as dishabituation stimulus followed by 1 min break. The blue square represents the aversive ultraviolet light. (B) Summary of seven experiments of brain activity recorded from ¾ head preparation (inset) during habituation and dishabituation. Note that the responses to the ultraviolet light remain almost constant throughout the experimental blocks. The activity level is shown as the sum of the units counted during the 5 s post-stimulation, represented as percentage of the initial response. The error bars represent the ±s.e.m.

Fig. 5 depicts brain activity measured from ¾-head preparations (*n*=7) during habituation to weak 0.5 s vibration stimuli, followed by 1 s of aversive UV-light stimulus that induced dishabituation. The learning protocol consisted of three sessions: the first session included approximately 40 repetitions of the stimulus (with a 30 s inter-trial interval), while the subsequent two sessions included about 20 stimulations each. A clear habituation and dishabituation process was observed in all three sessions. It is noteworthy that there were no significant differences between the measured responses to the UV light after each habituation session. Therefore, in addition to dishabituating the response to the vibration stimulus, the measured activity evoked by the UV light also serves as an important indicator that the reduction in the response to the vibration is not the result of a decrease in the suction electrode sealing or unrelated weaknesses of the nerves.

### Exploring the modalities and functioning of distinct brain regions

**Fig. 6.**
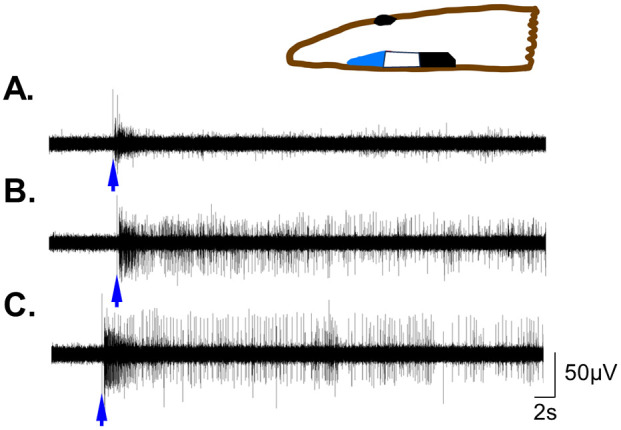
**Functioning of distinct brain regions along the brain tissue in a half head preparation.** Inset: the brain tissue was segmented into three sections, using the eye as a reference point. (A-C) Representative recordings of the evoked response induced by 0.5 s ultraviolet light (blue arrows), from each of the three different regions (indicated by the colors blue, white and black in the inset). (A) example from the anterior region (blue). (B) example from the region posterior to the eye ((white). (C) recording from the posterior region of the brain (black).

To demonstrate the ability of the recording technique to differentiate between functions of various brain regions, the responses to a range of stimuli (liver juice, vibration, UV light and touch) were tested in three areas along the exposed brain tissue in half-head preparations (Fig. 6). The brain was divided lengthwise into three regions, using the eye as a reference point (Fig. 6 inset). The first region extended from the eye to the anterior part of the brain. The second region was located below and posterior to the eye, while the third region included the posterior part of the brain, away from the eye. Shifting the electrode's position along the exposed brain tissue revealed distinct differences both in the brain activity pattern and responses elicited in each region. The anterior part was characterized by small amplitude units, almost no spontaneous activity and although responding to all stimuli, the evoked response in this area was very weak compared to the other regions (Fig. 6A). Both the middle and posterior regions displayed robust evoked activity for all stimuli (Fig. 6B,C). The recorded units’ amplitude was distinct for the different areas and increased as the recording electrode was moved posteriorly farther from the eye. In comparison to the posterior area, amplitudes from the middle area were more varied and the spontaneous activity slightly stronger. It should be noted that all experimental recordings presented here were carried out from the midline area.

### Simultaneous recording from two separate brains of a double-headed worm

**Fig. 7.**
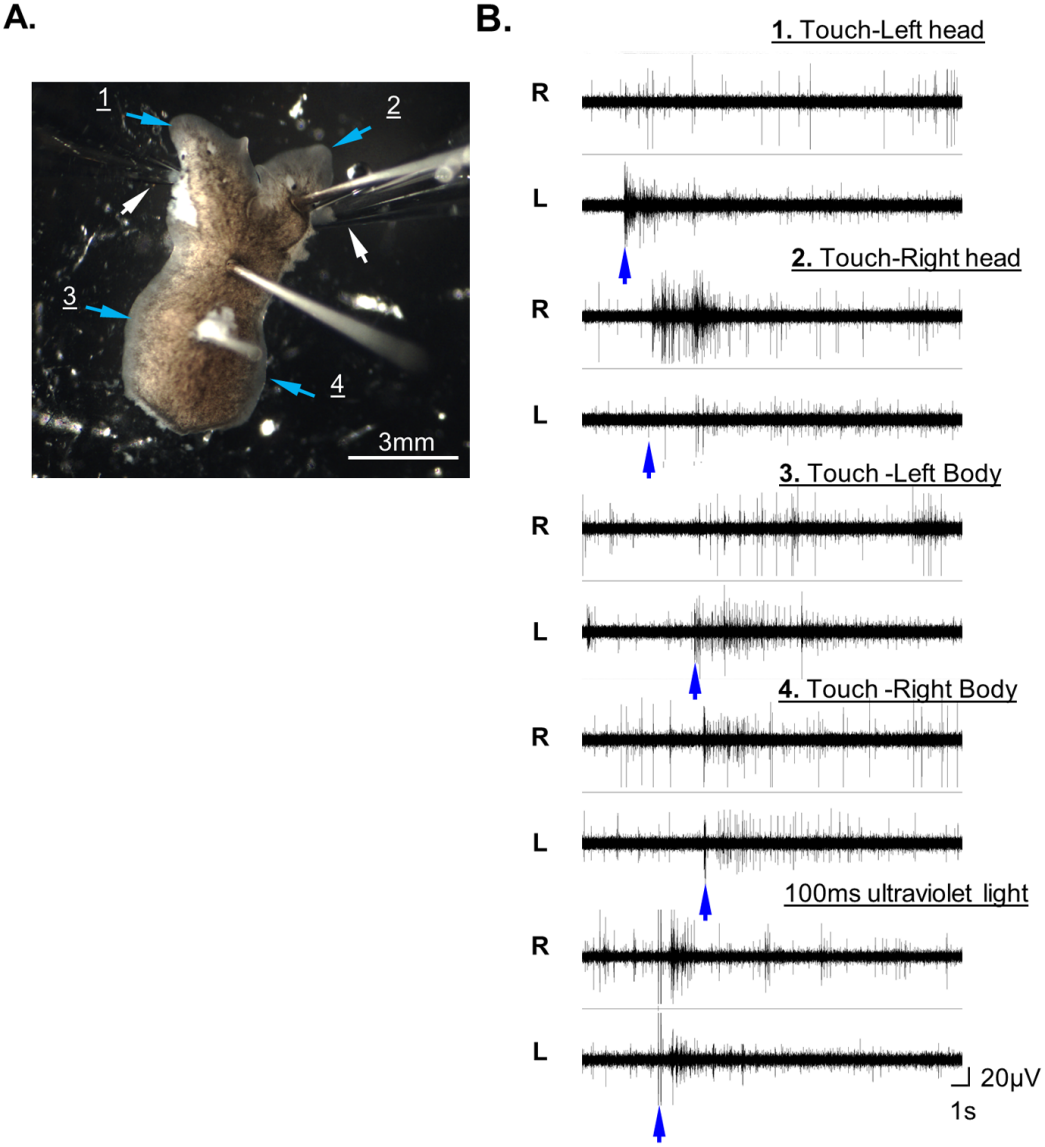
**Simultaneous recording from both brains of a two-headed worm.** (A) The preparation of the two-headed worm (*Dugesia* sp.) with suction electrodes attached to each opened ¾ heads preparation (white arrows). The worm was restrained with pins and its body was shortened by making a cut posterior to the heads. (B) The recorded responses from each brain (R=right, L=left) to manual touch on different sides of the common body (according to the numbers and light blue arrows in ‘A’. The two recording traces at the bottom of the panel depict the responses to 100 ms projected ultraviolet light on both heads. The blue arrows indicate the onset of each stimulus.

One of the exceptional experiments that can easily be done with planarians is to investigate the interaction between two separated brains that share a common body. Here, we show the feasibility of simultaneous recording from both brains. The planaria was restrained by pins and reduction of the body size by cutting behind the head (Fig. 7A). When UV light was projected on the entire planaria, it triggered a strong activity response in both brains. However, a directed manual touch on one of the heads’ margins elicited a response only from the brain associated with the touched head. Touching the sides of the body resulted in a stronger response in the ipsilateral brain than in the contralateral one (Fig. 7B).

### Testing linalool as an efficient anesthetic substance for electrophysiological studies in planarians

**Fig. 8.**
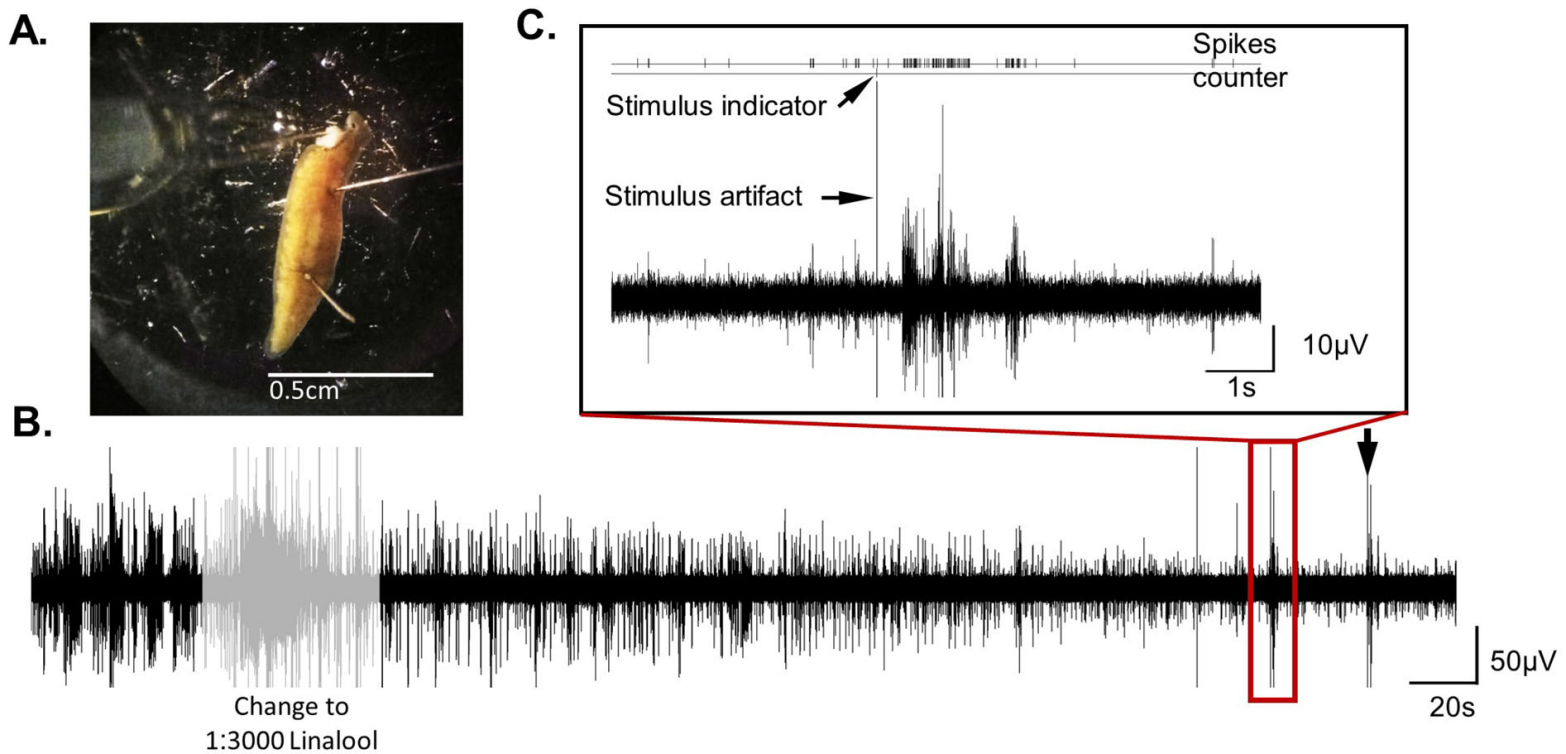
***in-vivo* extracellular recording from an intact worm that was “sedated” by recording solution that contained linalool.** (A) The ∼1 cm worm with intact body and half head cut, restrained with combination of pins, and linalool in concentration of 1:3000 that provides partial immobilization and maintained the brain activity. (B) Representative recording segment that contains spontaneous neuronal activity followed by bath solution exchange to linalool 1:3000 and evoked response to two 1 s projected ultraviolet light (30 s inter trial interval, red rectangle and black arrow). (C) Enlargement of the area around the first light stimulus labeled with red rectangle in ‘B’.

We evaluated the efficiency of linalool as a potential anesthetic agent for electrophysiological experiments based on three criteria: (1) complete immobility, (2) reversibility and minimal side effects, and (3) presence of neural activity under anesthesia. Worms that were exposed to linalool at a concentration of 1:1000-1:2000 became fully immobilized within a few minutes, even under the influence of aversive UV light. Their bodies stayed relaxed, exhibiting minimal bending, twisting, and pharynx ejections. The effect was reversible, with the worm regaining mobility after several minutes in the clean maintenance water. However, under a state of complete immobilization (with no behavioral response, even to aversive UV light), linalool abolished brain neural activity. Yet, by using reduced doses that did not lead to complete immobilization but maintained part of the neuronal activity, combined with the use of pins, we were able to restrain an ∼1 cm intact *Dugesia japonica* (Fig. 8A). Without the anesthesia, the worm would have been able to tear itself from the pins. Fig. 8B and C show the extracellular recording before and then under a sedation condition by the application of a 1:3000 linalool solution. Switching the recording solution to saline with linalool quickly reduced the amplitude of the units by about a half, and also decreased the average activity (Fig. 8B). However, it still allowed for the collection of quantitative and qualitative data on the neural response to stimulus such as the aversive UV light (Fig. 8C).

## DISCUSSION

Planaria has a number of features that position it as potentially unique and significant animal model for research in the field of neurobiology (Pagán, 2014). However, in contrast to its role in developmental biology and regeneration research where it has become a leading model, its contribution to neuroscience is marginal. The ability to monitor the neuronal activity is essential for any animal model in neurobiological research. Yet, as for the freshwater planarian, there are only a few previous reports of electrophysiological recording of electroencephalogram (EEG) waveforms (Aoki et al., 2009; Freiberg et al., 2023) and extracellular recording of the ocellar potential (Azuma et al., 1994; Brown and Ogden, 1968). The approach presented here is a straightforward, fast, and easy way to reach and record multiunit extracellular data from the tiny and fragile planarian brain. This work is a proof of concept and a first step of characterizing the neurophysiological basis behind the planaria behaviors. We hope that the approach presented in our work will enable a variety neurophysiological research in future.

### The implementation of recording from semi-intact head

The semi-intact head preparation is both easy to perform and highly efficient, enabling high-quality recordings of evoked and spontaneous neuronal brain activity from any brain region. Recording from the half-brain preparation reveals distinctive and clear responses to varied types of tested stimulations. However, it is worth to mention that it had been found that worms with half head, head splits, or incomplete regeneration of decapitated head, exhibit a reduction in behavioral assays related to seizure-like movements induced by cocaine or cytisin (Pagán et al., 2020, 2013). Therefore, in certain types of experiments, it may be preferable to use the ¾ head preparation, where the brain is left almost intact. The recording approach could also be relevant for investigating the longitudinal nerve chords. For example, that will allow to study the neurophysiological basis of the interesting intact-like behavior which display by headless worm fragments (Le et al., 2021).

### Restraining the worm by dissection pins and linalool as anesthetic agent

Restraint of the worms was done with fine dissection pins and decapitation that reduced the worm's body strength. This method proved to be effective and allowed durable and fine recording for more than 1 h, even in experiments where aversive stimulation that induce strong contractions was applied repeatedly. However, durable extracellular recording from intact large worms requires stronger restraints and immobilization. Moreover, fine intracellular recording, requires complete immobilization, even of internal body movements. Here, we evaluated linalool as an anesthetic for electrophysiological experiments. There are indications that the planarians’ glutamatergic and cholinergic neurotransmission shares similarities with those of mammals (Nishimura et al., 2010; Rawls et al., 2009). Therefore, it is possible that linalool's anesthetic effects are induced through inhibition of glutamatergic signaling and modification of cholinergic neurotransmission in the neuromuscular junction (Batista et al., 2008; Elisabetsky et al., 1995; Re et al., 2000). Linalool induced complete immobility that was reversible. However, in an anesthetized state of complete immobilization, no brain activity is discerned. Though, the combination of doses that induce partial immobilization but partially preserve neural activity, together with mechanical restraint by fine dissection pins, allowed extracellular recording experiment from ∼1 cm intact worm (Fig. 8). Perhaps a less active and vigorous species such as *Schmidtea mediterranea* (Blackiston et al., 2010) would be easier to restrain while recording from an intact large, partial immobilized worm.

### Differences in the recorded activity from three regions along the brain

Moving the recording electrode along the exposed brain tissue in the half-head preparation (Fig. 6) revealed clear differences in the spontaneous and evoked activity between the regions that were defined in relation to the eye position. In contrast to the anterior part of the brain, the area behind and posterior to the eye revealed a strong spontaneous and stimulus evoked response. This area includes the optic nerves, which project posteriorly from the optic chiasm, likely along with axons from other sensory modalities (Chan and Marchant, 2011; Inoue et al., 2015; Scimone et al., 2020). Therefore, our electrophysiological findings in conjunction with the histological studies, imply that this part of the brain serves as a hub for sensory integration. Another variation observed along the brain was the appearance of larger units as the recording site was moved towards the back of the brain. The origin of these large units might be the ventral nerve cord, which connects to the brain posterior to the eye and becomes more dominant towards the back of the brain.

### Continual application of UV light or mild vibration leads to sensory adaptation

The neuronal activity evoked by a 5 s exposure to UV light or mild vibration revealed a pattern of sensory adaptation for both stimuli. However, the UV light elicited a much stronger response, and at the end of the stimulus, the spontaneous activity remained at a higher sensitization level compared with the baseline prior to the stimulus (Fig. 4A), which is expected given that planarians are highly light-aversive. The response to vibration returned to the baseline level observed prior to stimulation. These response patterns illustrate the efficacy of using these two types of stimuli in learning protocols, such as habituation to a vibration stimulus combined with dishabituation by intense UV light. Using these two stimuli can also be suitable in the context of conditioned learning, where the UV light serves as the unconditioned stimulus and the vibrations as the conditioned stimulus.

### Distinct patterns of neuronal responses to electric shock, UV light, and vibration

Each of the three main tested stimuli, the UV light, electric shock and vibration revealed a distinct profile of evoked activity. The UV light induces robust activity response but with a latency of approximately 200 ms from the onset of the stimulus (Fig. 3E,G and Fig. 4B,D). At least part of the latency can be attributed to the ocellar potential latency, previously described by Brown and Ogden (1968) in addition to other factors that contribute to delays in sensory information flow, such as nerve conductivity, synaptic transmissions, and activation thresholds. The bath temperature and light intensity were more or less the same in all experiments and the only variable parameter was the stimulus duration. As reported by Brown and Ogden (1968) for the ocellar potential latency, our examination also revealed that the duration of the light stimulus had no apparent effect on the latency, even when the stimulus duration was substantially shorter than the latency time (50 ms, data not shown). The light stimulus was in the range of UV A (∼390 nm) and therefore, in addition to the sensory information from the eyes, the measured response might also incorporate signals from the extraocular sensing system. In fact, it is plausible that the majority of the recorded neural response is associated with extraocular receptors, which have a sensitivity spectrum more aligned with the UV-light stimulus used in this work (Birkholz and Beane, 2017; Shettigar et al., 2017), while the ocular receptors responsive spectrum is around 500 nm (Brown et al., 1968). The activity profile induced by the electric shock was long pause in the spontaneous activity for several hundred milliseconds (Fig. 3B,D). The electric stimulus was given superficially through the bath water and is not targeted to specific body area (Fig. 3A) and the evoked activity from the light stimulus is unaffected by that pause (Fig. 3F,G). Therefore, it seems that the two aversive stimuli do not activate the same neuronal pathway and that pause might be a result of synaptic vesicle depletion or maybe caused by suppression mechanisms in that specific pathway, similar to those observed in other models in response to nociceptive receptors (Reho et al., 2022). In some of the experiments the pause ended with elevated activity compared to the previous activity, but that evoked activity was abolished after several trials (Fig. 3B,C,D). It resembles habituation and is likely not a result of electrode polarization, because changing the polarity does not change the response and the decay. In addition, there is a silence for about a second after the electrical stimulation, and even when there is no evoked activity (Fig. 3D) the worm keeps responding to the shock with contractions, possibly from muscle spasm. Unlike the UV-light stimulus, the vibration stimulus elicits a response with inconsistent latency. It might be that the latency is a result of slower recruitment of sensory inputs that activate the recorded brain area and changes in the sensitivity of the sensory receptors and neurons. Pauses in activity and delays in response are attributed to the inherent conductivity, synaptic fatigue and response properties of the involved neurons, as well as more complex mechanisms within the neural circuit. (Brill et al., 2014; Mendell, 1966; Moscato et al., 2019; Tengölics et al., 2019). Investigating these processes in a simpler animal model, such as the planarian brain, could potentially aid in deciphering and comprehending their function in the processing of sensory information.

### Monitoring the brain activity during learning process

The ability to study the well-documented learning behaviors of planarians at the neuronal activity level is a significant step toward establishing the planarian as a model for neurobiology research. We successfully employed the developed recording technique and the defined stimulation procedures to investigate brain neuronal activity during the simple learning process of habituation to repeated vibration stimuli followed by dishabituation induced by single UV light exposure (Fig. 5).

### Differential responses recorded from the brains of a two-headed worm

Studying how two brains function in a shared body is an excellent example of an experimental approach yielding reproducible and manageable research available only in the planaria model. In the example presented in Fig. 7, we simultaneously recorded from two heads while applying tactile stimulation to various body areas. The results demonstrated that the brain's responses were contingent on the stimulated area. Tactile stimuli on the body's side elicited a much stronger response from the ipsilateral brain, and tactile stimuli around the heads induced a clear response only from the stimulated head's brain. These results validate the feasibility of the procedure and suggest that the neuronal network was adjusted and shared between the two brains.

## MATERIALS AND METHODS

### Experimental animals

All experiments were conducted on planarians*, Dugesia japonica* 1-1.5 cm long, except the recording from two-headed planarian which was done with *Dugesia* sp. from a colony established from worms collected from the upper course of the Jordan River, Israel. All planarians were kept in glass containers filled with aged tap water with an initial pH value of 8.2-8.3. However, subsequent pH checks revealed a value around 7.5, probably due to their biological effect on the water pH. It also been suggested that they are able to control the pH in the closed environment of the container (de Oliveira et al., 2018). The containers were stored in an incubator at 16°C in continuous darkness. The worms were fed mashed organic chicken liver once or twice a week, and were starved for at least 3 days before using them for recording experiments.

### Experimental preparation

A semi-intact preparation was prepared on a dissection cooling plate under a dissection microscope, with Surgical Blade No 11. To expose the brain, the head tissue was cut in longitudinal planes, depending on the area of interests (Fig. 1). For half-head preparation, the head was cut a bit to the side of the middle line, just before the eye (Fig. 1A,B). If the head is cut exactly in the middle and not a bit to the side, there is still nonneuronal tissue covering the brain, which prevents a good connection between the brain tissue and the recording suction electrode. The second preparation tested in this work was a ¾ head, where the head was cut along the outer side, in line with the eye (Fig. 1A). The brain tissue is located in the ventral region of the head and can be identified as a slightly transparent tissue that has a distinct texture compared to the tissue above it. To restrain the worm during the recording, it was pinned with fine dissection pin (0.1 mm). The pins should be as fine as possible to minimize injury to the head and decrease the possibility that the worm will tear itself off the pins. Also, to weaken the worm's ability to tear itself from the pins during recording, the planarian head was separated from the body by a cut in the neck (Fig. 1B). In addition, to suppress motility in order to facilitate precise directing of the electrode to the desired brain region, the planarian head fragment was transferred to a small glass bowl with ice-cold extracellular solution for 1-2 min before transferring to the recording bath (35 mm petri dish) where it was pinned to the SYLGARD 184 transparent silicone bottom (Fig. 1D). The recording bath was also been filled with ice-cold extracellular solution just before placing the worm. This further reduced the worm's movement, making it easier to attach the recording electrode. In most of the recording sessions the spontaneous activity was stronger at the beginning and decreased over the course of the experiment. In an attempt to start all experiments at a similar level, we waited 5-10 min after the connection of the suction electrode and let the worm rest before starting the recorded experiments.

Fig. 1C presents another feasible approach (not used for the experiments presented in this work), where we completely exposed the brain by dorsally peeling the skin and tissues above the brain (Koopowitz, 1975; Wang et al., 2016). However, the loosely structured brain of the planarian tends to lose its form without the surrounding tissues, and the ventral muscles tend to contract and bend the preparation. As noted by Koopowitz (1975), the contraction and bending of the preparation can be reduced by cutting off the marginal rim of the worm. However, the rim around the head possesses the main sensing region and therefore the procedure might not be suitable for experiments related to sensing. This dissection approach is very challenging and time-consuming, but could provide a more accurate characterization of the recorded brain regions, and may well be more convenient for isolated brain preparations and intracellular recording.

The two-headed planarian preparation was generated by midsagittal cut of normal worm's head and body until just above the pharynx. To prevent the two cut sides from reconnecting, we first placed the operated worms in a refrigerator for several hours. The cold temperature allowed the wound to slowly close with minimal movement of the heads. Additionally, during the first few days after surgery, we closely inspected the worms and if necessary, we renewed the midsagittal cut to maintain the separation between the heads. The worms were considered ready for experiments after a few weeks, when the renewed sides of each head were indistinguishable from the original sides in terms of size, pigmentation, and eye shape.

### Extracellular recording

Extracellular multi-unit recording obtained by a suction electrode made from a glass micropipette (glass capillaries, WPI 1B150-4) that was cut and fire polished to an open of 70-100 µm containing a coated platinum-iridium wire (Bare diameter 127 µm, A-M System. 778000) uncovered at the tip. The reference electrode was made by uncovering a few centimeters of coated platinum-iridium wire and rolling the uncovered wire on the glass capillary to create a coil with a large contact surface (Fig. 1D). No difference was observed when the reference electrode touched the worm or was inserted into the tissue, instead of placing it near the head. It is worth mentioning that during the development of the protocol, we noticed that the UV-light stimulation creates a large voltage artifact when we used silver wire for recording. Therefore, the commonly used silver wire is not recommended for experiments involving light stimulation, as it is sensitive to light. This sensitivity can lead to the production of artifacts that might be difficult to differentiate from genuine neural activity, particularly in the case of local field potential or EEG signals (Liu et al., 2018; Moody et al., 1969; Van Peski et al., 1969).

The neuronal activity recorded by differential amplifier (A-M Systems Model 1800) connected to CED – Power 1401-3A data acquisition interface, operated through CED Spike2 software. The various stimulation protocols and event tagging were carried out using the Master-8 pulse generator. The signal sampled at 20 kHz, differentially amplified (×10k) and was bandpass filtered at 0.3-1 kHz which provided good signal to noise ratio from all the recording sessions. Despite this bandpass-filter parameters do somewhat reduce the signal's amplitude and might slightly alter the unit's waveform, though it was precise enough for the spike counting analysis performed in this work and even for spike sorting analysis (not shown). In about half of the recording session, as can be seen in Fig. 2, even when bandpass-filtered at 0.3–10 kHz, a distinct signal can be captured, enabling the recording of a clear extracellular spike waveform characterized by a slightly prolonged duration of 3-4 ms (Fig. 2B).

### The stimuli configuration

Five types of stimulation were applied:

1. Light aversive stimulus (Paskin et al., 2014) made from conventional laser pointer adjusted and connected beneath the recording stage and projected long wave UV-light (380 to 450 nm, Peak at 388), from below through the transparent silicone bottom (Fig. 1D).
2. Vibration stimulation made from tatoko DC Coreless Motor Built-in Vibration Waterproof, 1.5-3 V 8000-16000RPM, connected to a plastic rod placed close to the head of the planaria (Fig. 1D).
3. Electrical stimulation - 1 mA DC electric stimulation delivered through ISO-Flex Stimulator (A.M.P.I) connected to a bipolar coated platinum-iridium wire which is less prone to polarization (Figs 1D and 3A). The electrode wires were positioned on both sides of the worm, close to, but not touching its body (Fig. 3A).
4. Tactile stimulation - a gentle touch on the skin using a fine plastic filament, applied manually.
5. Chemical stimulation, ‘liver juice’ made by vortexing a liver mash in a tube with the recording solution followed by centrifugation. The supernatant was then used as the ‘liver juice’. Application of the ‘juice’ (∼30 nanoliters) near the worm's head was done through a nanoliter injector (Nanoject II from Drummond Scientific Co.) and induced a clear evoked response with a delay of 1-2 s.

### Recording solution

Since there is no established protocol for planaria extracellular recording solution, we adopted HEPES-buffered saline used for electrophysiological recordings from the freshwater snail *Lymnaea stagnalis* (Pirger et al., 2021). The saline contains: 50 mM NaCl, 1.6 mM KCl, 3.5 mM CaCl_2_, 2.0 mM MgCl_2_, 10 mM HEPES. Originally the pH for *Lymnaea* solution was 7.9 and we adjusted it with NaOH to pH 7.4 which is the commonly used pH for planarian cell culture medium (Schürmann and Peter, 2001). This produced good recording quality and meaningful results, though we cannot be certain that this solution is the most suitable for planaria electrophysiology. In previous works, Brown and Ogden (1968) used cell culturing solution at pH 7.4 and Azuma et al. (1994) used 5/8 Holtfreter solution at pH 7.6, for extracellular recording of the planarian ocellar potential. The two key parameters are the osmolarity and pH. The saline osmolarity provides the conductivity for the electrophysiological recording and should reflect the ionic composition of the internal extracellular environment. The osmolarity and pH was in the range of previously used saline for planaria cell culturing (Schürmann and Peter, 2001). We can assume that the rise in salinity (compared to the water used for the planarians maintenance) effects the semi-intact planarian state and behavior. Though, when placing intact *Dugesia japonica* into the Lymnaea saline they exhibit mild twisting for a short period and tendency to aggregate (which might indicate for stress); but they survive and behave quite normally for at least 24 h (the duration of the observation).

### Anesthetizing the worm

Linalool 97% (Sigma-Aldrich, catalogue number L2602) was tested for its efficiency as an anesthetic agent (Goel et al., 2019; Hall et al., 2022). Linalool was mixed within the planarian maintenance water at a ratio of 1:1000-1:2000, and the worm behavior and response to aversive UV-light was monitored.

We also tested linalool as an anesthetic agent for the use in electrophysiological experiments. During the recording session, the recording bath solution was changed to linalool that was mixed with the recording solution at a ratio of 1:3000. It is important to note that linalool evaporates slowly. Thus, when using it for anesthesia, the container housing the worms should be covered. In the case of the recording bath, care should be taken to refresh the recording solution during the experiment.

### Data analysis

Data analysis was conducted offline using CED Spike2 software and Excel. Activity was quantified by counting the spikes that rose above a threshold (∼2 standard deviations of the baseline noise). For a clearer view of the activity pattern, a smoothing function (1 ms moving average) was applied offline, to all displayed recording traces, except for the recording shown in Fig. 2. It should be mentioned, that this offline process significantly reduced the amplitudes of the spikes by tens of milli volts, which is the main reason for the differences in amplitude seen in Fig. 2 compared to the other figures. Statistical analysis of repeated measures ANOVA for the results shown in Fig. 4 was performed using JMP. All the single examples showcase in this study represent consistent results that have been obtained from at least, three biological replicates, except the recording from the two- headed worm which was examined in two worms.
